# Signal Processing for Metagenomics: Extracting Information from the Soup

**DOI:** 10.2174/138920209789208255

**Published:** 2009-11

**Authors:** Gail L. Rosen, Bahrad A. Sokhansanj, Robi Polikar, Mary Ann Bruns, Jacob Russell, Elaine Garbarine, Steve Essinger, Non Yok

**Affiliations:** 1 Electrical and Computer Engineering Department, Drexel University, Philadelphia, PA, USA; 2 School of Biomedical Engineering, Science, and Health Systems, Drexel University, Philadelphia, PA, USA; 3 Electrical and Computer Engineering Department, Rowan University, Glassboro, NJ, USA; 4 Soil Science/Microbial Ecology, Pennsylvania State University, University Park, PA, USA; 5 Biology Department, Drexel University, Philadelphia, PA, USA

## Abstract

Traditionally, studies in microbial genomics have focused on single-genomes from cultured species, thereby limiting their focus to the small percentage of species that can be cultured outside their natural environment. Fortunately, recent advances in high-throughput sequencing and computational analyses have ushered in the new field of metagenomics, which aims to decode the genomes of microbes from natural communities without the need for cultivation. Although metagenomic studies have shed a great deal of insight into bacterial diversity and coding capacity, several computational challenges remain due to the massive size and complexity of metagenomic sequence data. Current tools and techniques are reviewed in this paper which address challenges in 1) genomic fragment annotation, 2) phylogenetic reconstruction, 3) functional classification of samples, and 4) interpreting complementary metaproteomics and metametabolomics data. Also surveyed are important applications of metagenomic studies, including microbial forensics and the roles of microbial communities in shaping human health and soil ecology.

## 1. INTRODUCTION

Currently, the complete genome of an organism is obtained through 1) isolating and culturing the organism to obtain sufficient DNA mass, 2) extracting and amplifying DNA, 3) sequencing the genomes, 4) assembling them, and 5) finally annotating genes and regulatory elements. This process breaks down at the first step for organisms that cannot be cultured. Given that >99% of microbes cannot be cultivated in isolation [1], this traditional approach has vastly constrained our ability to study microbial genomes. New approaches propose to start at step 2 and sequence as much as possible of the DNA present in a sample, but such sequencing is slow with classical methods.

PCR-based techniques that can identify ribosomal RNA show what species are present in a sample. However, isolation and culturing of an individual species has conventionally been required to obtain its genome sequence. One of the most compelling advantages of metagenomics is avoiding the need to isolate and culture individual organisms. When people think of cultivating microbes in culture, they typically imagine bacteria growing on a dish with agar. There are indeed a number of bacterial species that grow easily in such cultures, such as *Escherichia coli*. Not coincidentally, such bacteria are the most well-studied and the first to be sequenced. However, the vast majority of bacteria. Bacteria often require specific growth conditions that are either difficult to achieve in a laboratory or even unknown. For example, Legionella pneumophila, the bacteria that cause Legionnaire's Disease, were not cultured until 6 months after the original outbreak of the disease. This was despite an intense effort by CDC scientists [2]. A recent study suggested that over 60% of the bacterial species found in the amniotic fluid of women with preterm births were from uncultured or difficult-to-culture species [3]. Culture-independent techniques have found that half or more of the bacteria in the human mouth are uncultured species [4]. Overall, past work has shown that perhaps 85% or more of total bacterial diversity consists of uncultured species [5]. Metagenomics provides the only way to obtain gene sequences for these otherwise hidden organisms.

Fortunately, the recent advent and application of high throughput next generation sequencing methods have enabled a large increase in productivity [6, 7]. This allows the decoding and assembly of multiple genomes from multiple species in communities. This now becomes the field of metagenomics, where scientists must now think on a broad-scale [8, 9], shifting their focus from “How does one organism work?” to “Who all is here and what are they doing?”

This shift is not the only challenge facing biologists in the emerging era of metagenomics. The increased complexity of the data poses challenges in assembling, annotating, and classifying genomic fragments from multiple organisms. Complications also stem from the difficulty of assembling, annotating, and classifying the short sequence fragments typically obtained with next-generation sequencing methods. So, novel computational methods are needed to address these issues and the massive amounts of sequence data that have become available through recent technological advances.

Signal processing and machine learning disciplines are well-equipped to solve problems where background noise, clutter, and jamming signals are commonplace. Hidden Markov models (HMMs), originally popularized for speech processing, have been used for over a decade for gene recognition [10], and it has been found that many techniques used in speech and text mining can now be applied to biology. Metagenomics allows the classification of millions of organisms and their genes, including identifying particular community differences and markers. Supervised and unsupervised machine learning methods, linear classifiers, advanced Bayesian techniques, etc. are all promising to advance rapid annotation and comparison of samples. In this paper, we survey the potential and utility of new methods in metagenomics, which are already revolutionizing the field of bioinformatics. In doing so, we emphasize how these approaches allow us to identify the taxa from which sequenced fragments originate. Furthermore, we highlight how tools for functional annotation have shed light on the coding capacities of natural bacterial communities, focusing on the potential harmful or beneficial consequences of these microbes from a human perspective.

## 2. EMERGING BIOLOGICAL STUDIES IN METAGENOMICS

It is important to highlight the biological objectives of metagenomic studies. In this section, some of the more exciting and potentially useful applications are reviewed.

### 2.1. Human Health

In the human gastrointestinal tract, microbes outnumber human cells by 10 to 1, and approximately 100 trillion live in the gut alone [1]. Microbes symbiotically perform functions that humans have not evolved, including the extraction of calories from otherwise indigestible components of our diet, and the synthesis of essential vitamins and amino acids. It has been hypothesized that an imbalance in microbial health can cause obesity [11], and methods are needed to determine what microbes and/or metabolics contribute to a microbial community's behavior.

The National Institute of Health has extended an initiative, entitled The Human Microbiome Project, to examine microbes associated with health of several areas of the human body [12]. These include: 1) our gastro-intestinal (GI) tract [11, 13-16], 2) the oral cavity [17, 18], 3) the nasal cavity/lung, 4) skin [19], and 5) genital regions [20]. GI-illnesses and tooth decay have loosely been linked to “bad” build-up of bacteria that cause cavities [17], but the make-up of these bacterial communities needs extensive study. The taxonomic and functional characteristics of these microbes can then be used to decipher the mechanisms behind potentially harmful or beneficial activities of human bacterial associates. The results of metagenomic analyses may contribute, for example, to improving the formula and use of mouthwash [21].

### 2.2. Soil Fertility

Microbial soil communities are highly diverse [22], consisting of many undescribed bacterial lineages [23]. It has been shown that some soils are more capable than others of supporting growth of healthy plants, and that many desirable soil properties are correlated with microbial composition in the soil [24]. Soil microbial communities have been implicated in the suppression of plant pathogens [25], and breakdown of pollutants [26], which favor agricultural productivity. It is hypothesized that degraded soils with low microbiological diversity suffer from an imbalance of nutrients and cannot suppress plant pathogens [24]. This suggests that humans could stimulate soil microbial processes that assist plant growth by replenishing nutrients favoring beneficial microorganisms. Greater knowledge is needed of how agricultural management practices induce shifts in soil microbial community composition and function [27]. Metagenomic studies could lead to understanding how changes in soil microbial communities influence long-term agricultural sustainability.

### 2.3. Forensics

The anthrax scare of 2001 highlighted the need for microbial forensics. The Bacillus anthracis spores found in the mailed envelopes were related to the Ames strain, commonly used in research in over 20 laboratories [28, 29]. Since the Ames strain was created, unique point mutations arose separately in distinct populations grown in separate labs. Because the anthrax-laden envelopes contained billions of spores, many of these envelopes harbored mutations that further distinguished them from existing lab populations. Since scientists did not initially know where these mutations had occurred, elucidating the origins of this anthrax strain required a large amount of genome-wide sequencing and analyses to generate sufficient data for evolutionary reconstruction [29]. Metagenomics techniques were crucial in obtaining the diversity of mutations within the envelopes' samples [30].

Recent applications of metagenomics to studies of ancient DNA [31, 32] may benefit the field of forensic science. For example, to study the genome of the extinct wooly mammoth, DNA was extracted from well-preserved mammoth remains and sequenced using the Roche/454 method of pyrosequencing [33]. Although a considerable proportion of sequence reads came from the genomes of other organisms, approximately 50% were closely related to the elephant genome, suggesting that the authors had successfully sequenced mammoth DNA from 28,000 year-old remains [34]. A similar approach has also been used to study the genomes of extinct Neanderthals [35], and may be applied to the study of human remains or environmental samples from crime scenes. Such a technique can offer the opportunity to identify victims, to detect DNA from a suspect, or to match the microbial profiles from samples at the crime scene with those observed in association with an identified suspect. These methods may also enable detection of air-borne pathogens within indoor facilities [36] or soil in outdoor environments [37, 38], an area of special concern in the attempt to prevent effective bioterrorism [28].

## 3. METAGENOMIC TECHNOLOGIES

The first step of any metagenomics study, is to acquire the data -- whether it be DNA sequences, specific genes, mRNA, or proteins. This first step is fundamental to the process, and is the assumption on which further analysis and comparison operate. Any technological limitation with the first step must be compensated for in subsequent analysis. 

### 3.1. DNA Sequencing

Traditionally, DNA has been sequenced using a chain-termination method developed by Fred Sanger *et al*. [39]. This method revolutionized genomics by being able to read (or identify the nucleotide bases of) complete genes. Since then, the method has been refined and it produces the average read-length of 750 basepairs (bp). However, this process requires several steps, with current instrumentation, and can only process 96 reads at a time, thus rendering this method extremely slow and costly [6, 40]. Recently, next-generation sequencing technology has emerged which can process millions of sequence reads in parallel, requiring only one or two instrument runs to complete an experiment. But this massively parallel approach comes at a price -- most next-generation technologies produce sequence reads much shorter than 750bp.

For example, the Roche 454 pyrosequencers can obtain 400K reads, each with an average length of 250 bp (a total of 100 Megabases per 7-hour run) [6]. Illumina sequencing-by-synthesis, on the other hand can deliver 36 million reads of average length of 35bp in 4 days (a total of 1.3 Gigabases per 4-day run) [6]. In the end, the throughput is similar, but the pyrosequencing method yields longer reads. Longer reads are likelier to yield uniquely identifiable sequences that are easier to BLAST [41] or to string-match to a database [7]. Because short reads miss some homologs found only in longer reads, doubt has been cast on the feasibility of short-read technologies [42]. Therefore, it is of current interest to show that metagenomic methods can overcome poor resolution of short reads using computational techniques.

### 3.2. 16S rRNA Detection

Instead of sequencing the DNA of an entire sample, which can be costly with traditional sequencing, a common approach is to restrict sequencing to taxonomically informative genome segments, such as those coding for highly conserved ribosomal RNAs. The 16S and 18S rRNA genes, with respective lengths of 1500 bp for prokaryotes [23] and 2800 bp for eukaryotes, encode RNAs destined for small subunits in ribosomes, the essential and universal sites in all cells where messenger RNAs are translated into proteins. Because these genes are so critical for proper cell function, they are highly conserved and reflect genetic variation among all life forms over evolutionary time. Sequence variations in these genes thus signify fundamental differences among phyla/divisions/genera/species. To obtain these sequences from complex mixtures of genomes, classical polymerase chain reaction (PCR) is used with primers complementary to the highly conserved regions of 16S rRNA [43-45]. Searchable databases for phylogenetic placement of new sequences are available in GenBank, RDP [46], while other models are based on shorter portions (500- bp or 400-bp) of 16S rRNA genes which are neither highly conserved not hypervariable and which have been used to distinguish various genus and species [47]. Recently, organism detection has moved to microarrays composed of 16S probes, which do not require long amplification steps [48-50].

### 3.3. Metaproteomic Technologies

In addition to meta* genomics*, other “omics” approaches hold great promise for deciphering complex mixtures. One emerging area is that of metaproteomics. Traditionally, scientists have been able to separate proteins from complex mixtures of cellular extracts using 2-D gel electrophoresis [51]. In the 90's, mass-spectrometry enabled rapid and highly sensitive protein identification [51]. In Schulze *et al*. [52], a mass-spectrometry (MS) method to analyze the protein complement of water containing organic matter from four different environments was introduced. Subsequent studies have used variants of MS approaches [53-55]. Although this article focuses on metagenomics, metaproteomics is discussed briefly in section 6.

## 4. GENOME-CENTRIC METAGENOMICS

Microbial community classification and comparison may appear at first as a daunting challenge. Yet, the problems are not too different from traditional signal processing applications. As in many applications, such as speech recognition, the first step starts with a vast amount of data. If the problem were posed -- “Given a set of acoustic waves from speech, decipher the words being said,” the solution seems distant at first. After decades of research on acoustic theory and speech processing, there is a rich theory describing how to segment the data and extract features followed by clustering and classification. A similar approach is extended to metagenomics. Fig. (**1**) illustrates the parallel between speech processing and metagenomics.

Metagenomics in its infancy has focused on two of three fundamental questions -- “Who is here?” and “How much of each is here?” [1, 56-58]. (With an emerging third question addressed in sections 5 and 6 -- “What are they doing?”). In early metagenomics project, such as the Venter Institute's Sargasso Sea project and Sorcerer II Global Ocean Expedition, 2 million sequence and 7.7 million reads were collected, respectively [59].

To even answer the “Who is here?” question, the analysis is complicated with a mixture of organisms. Remember, biologists traditionally culture an organism, so this question has not even been considered before. Usually, in single-genome analysis, DNA reads are all considered to be from the same genome, where each read can be matched to the **one** reference genome, and can therefore be thought as contigs (contiguous fragments) which form a scaffold. But now, in the environment, there are multitudes of genomes from a diversity of organisms, where the amount of each organism varies. Also, each DNA read can be from hundreds of *known* or millions of *unknown* genomes. A given environmental sample will have hundreds of thousands of organisms corresponding to billions, if not trillions, of basepairs -- and some organisms may only compose 0.01% of the sample. For example, it is known that pathogenic bacteria are present in our bodies at all times, but they are competing with healthy bacteria and are present in such small amounts, that it is negligent to our overall health. Usually, when the balance of “bad” to “good” increases, health problems arise. So one major question is -- if we gather a sample from the human gut, and a majority of the bacteria are probiotic *E. Coli*, how can we detect the few that are pathogenic? The near-10 million readers from the Venter expeditions, is just scratching the surface of all the diversity in the sea.

In signal processing, we usually think of capturing information in time -- that if there is a quickly changing (or high-frequency) signal, we need a higher sampling rate to detect it. In metagenomics, the case of sampling (or sequencing) is -- how well do you want to detect the “infrequent” signals/organisms? If one wanted to detect the top-5 organisms in a sample, it would probably be acceptable to undersample the environment because of high-redunancy of abundant organisms; compressive sensing techniques would be valuable here. But if the objective is to determine ALL organisms present, infinite sampling would most likely be needed. Biologists have stated that metagenomics samples can only be sampled and never fully characterized [1], and given prior knowledge about low-diversity, it has been hypothesized that some low-complexity environmental samples would need to be oversampled by 10 × to get a decent coverage of diversity [1, 42]. But to generalize this mathematically given different environments is still an open-problem, and metagenomics still needs its own Nyquist theorem.

To further quantify this to a metagenomics problem, we can formulate the data types associated with metagenomics. For example, it is well-known that DNA is composed of a discrete, finite alphabet, {*A,T,C,G*} [60], and therefore different discrete, word-like features can be formed. However continuous valued features can be generated from such data, such as the probability/frequency profiles of different *N*-mers. Also, there is the fundamental unit of the “gene”, and this can be used as a discrete feature and its frequency can be continuous.

The computational objectives associated with the “Who? How much? and What are they doing?” problems can be broken down into different categories. For the “Who?” question, a current problem is taxa-recognition which would be to classify reads into different hierarchical classes, such as top-level Kingdom, the mid-level Order, or even as specific as the type of strain. The difficulty in going higher and higher resolution, is that in biology the definitions become quite arbitrary and nonlinear on the genome-level. Some biologists are considering more genomic-definitions for defining taxa. The “How much?” problem is associated with the “depth” of the sampling, and obtaining a statistical confidence in the read-classifications. For example, with a particular error rate in classification, can we still say that the amount of reads classified do represent the true representation of a taxa in a sample? The emerging “What are they doing?” question has computational objectives on several different levels -- can individual genes be recognized from reads? This signifies the potential function of a sample. Also, once these genes are recognized, are they associated with pathways [61]? Another area, are what secondary structures are predicted and what genes are actually expressed in sample? -- which now goes into meta-proteomic and transciptomics.

To solve the “Which taxa and how much?”, there are vast amounts of unlabeled test data; very little labeled data is available to “train” on. Therefore, the genome fragment classification problem can be broken down into a) supervised *vs*. b) unsupervised methods [62].

The computational objective in this problem can be formulated in the following way: Given a feature vector x=[*x*_1_,*x*_2_,...,*x_N_*], obtained from the raw sequenced DNA, through some feature extraction approach, the learner *L*, is trained to recognize presence of one or more genomes in the set *G = g_1_,g_2_,…,g_M_*. In a supervised problem, the applicable labels for each **x** is available to *L*, whereas in an unsupervised problem *L* is simply asked to determine the clusterings within the data. Since the learner is not guided by the labels of the existing training data, unsupervised clustering is often a much harder problem. Going back to the speaker / speech identification problem: Having prelabeled data from, say 10 speakers, and asking the classifier to recognize each speaker based on the prelabeled data would be the supervised problem, whereas, providing all the data to an algorithm without labels, and telling to cluster the data into as many distinct categories as it finds would be the clustering problem.

The limitation regarding the availability of training data is also closely associated with the dimensionality of the data. When working with HMM for gene recognition, which are only 1000-2000 bp in length, researchers rarely venture past 5-mer feature sizes, but for whole-genome analysis, much greater feature sizes are needed [63, 64]. This poses huge problems for computing pattern recognition algorithms. For example, if one were to use the *N*-mer frequency profiles as features, the length of the feature vector grows very quickly (exponentially) with *N*. While most classifiers can handle feature vectors that are in the hundreds or even thousands of points, when the feature length reaches millions or hundreds of millions (4^9^, 4^12^, etc.), most popular classifiers become infeasible. Classifiers such as MLP, SVMs or other neural networks, that need to solve complex optimization problems (where feature sizes such as 4^9^) are near impossible, while simpler classifiers such as k-nearest neighbor - or even dimensionality reduction approaches (such as PCA) become unfeasible (working with a 4^12^ by 4^12^ matrix).

The problem is complicated more because unlike a standard classification problem, where *L* chooses only one element of *G*, more than one element of *G* may be chosen in the metagenomics problems. This can be true because multiple DNA reads maybe belong to different strains, or closely-related *G*. Also, in the case of horizontally transferred genes, similar sequence can be in unrelated *G*.

### 4.1. Supervised Taxonomic Classification

Supervised classification methods have traditionally been more popular, since unsupervised methods rely on intrinsic, possibly false, assumptions of the data. The disadvantage of supervised methods is the lack of sufficient data for training. Only a fraction of the species diversity exists in the current databases, and estimating diversity has been seen as unknowable as it is in constant change [65], making supervised approaches difficult to apply. However, as our knowledge of genomes expands, supervised methods hold promise to learn the data that will become available.

In this section, we review several methods in the following table:

**Table:** 

Features	Classifier	Published Method
Homology-based	Nearest-Neighbor	BLAST [41]
	Nearest-Neighbor & Last Common Ancestor	MEGAN [66]
Composition-based	Naïve Bayesian	Sandberg *et al*. [67]
		RDP classifier (16S sequences only) [46]
		Rosen *et al*. [64]
	Support Vector Machines	PhyloPythia [63]

#### 4.1.1. Homology-Based Approaches

Many current approaches align sequenced fragments to known genomes using homology [16, 42, 66, 68-72]. As mentioned in section 3.1, DNA is fragmented during sequencing so that the sequencer can “read” (or call the bases of) a relatively short length of DNA. Usually, the shorter the fragment, the shorter the time it takes to sequence, thereby driving next-generation technology. Short reads are generally not unique, thus yielding ambiguous classifications, and this has cast doubt about their applicability to metagenomics [42, 68, 72]. Therefore, when classifying sequences, an important aspect is to assess methods for these short-reads.

When the Venter Institute first shotgun-sequenced fragments from the Sargasso Sea, the natural first step was to BLAST these sequences against the comprehensive Genbank database [69, 73]. Although, the closest BLAST hit is often not the nearest neighbor [68]. Yet, without questioning the results, most metagenomic analysis relies on BLAST [16, 66, 70]. Only recently researchers have begun to analyze and compare the performance of BLAST for metagenomic datasets [42, 74]. Simply classifying genomic fragments based on a best BLAST hit will yield reliable results only if close relatives are available for comparison. While recently published MEGAN software relies on BLAST for analysis, it attempts to address this problem by classifying DNA fragments based on a lowest common ancestor algorithm (LCA) [66]. LCA allows fragments to generalize to a higher branch in the tree and not the nearest neighbor. Mavromatis *et al*. [75] show that homology-based approaches have lower specificity and hence are not very accurate. But, it has been shown that BLASTing all random sequence reads (RSRs) in a sample has comparable performance and can be faster and cheaper than extracting 16S sequences alone [74].

A notably relevant analysis demonstrates the drawbacks of using BLAST to identify short-reads from next-generation technology. For most metagenomics datasets to date, the significant BLAST hits only account for 35% of the sample [42]. Wommack *et al*. [42] take long read metagenomic samples and randomly chooses a shorter read within the larger one. The performance of BLAST nucleotide annotation is compared to BLAST for protein function classification using Clusters of Orthologous Genes (COGs). Short-reads retrieve up to 11% of the sample with correct BLAST hits and significance. They find that short reads tend to miss distantly-related sequences and miss a significant amount of homologs found with long reads. Therefore, improving short-read (less than 400bp) taxonomic and functional classification are open problems.

#### 4.1.2. Composition-Based Approaches

Besides homology, there are many sequence-composition based approaches [46, 63, 64, 67, 76-84]. Compositional approaches use features of length-*N* motifs, or *N *mers, and usually build models based on the motif frequencies of occurrence. Intrinsic compositional structure has been instrumental in gene recognition through Markov models [10] and in tandem repeat detection [60, 85]. In [76-78, 80-84], evolutionary and classification methods are based on di-, tri-, and tetra-nucleotide compositions, which soon lead researchers to look at longer oligos for genomic signatures [79]. Wang *et al*. [46] use a naive Bayes classifier with 8 mers (*N* mers of length 8) for 16S recognition. Researchers have since investigated ranges of different oligo-sized frequencies, with the initial pioneering work and the first naive Bayes implementation by Sandberg *et al*. [67]. McHardy *et al*. [63] found that 5mer and 6mer signatures worked the best for support vector machine (SVM) classification, but they concluded that accurate classification only occurs for read-lengths that are ≥ 1000bp. Sandberg *et al*. were able to obtain over 85% genome-accuracy performance for 400bp fragments using 9mers on a dataset of 28 species. Rosen *et al*. [64] took this further to show that the method can achieve 88% for 500bp fragments, but more impressively, it can achieve 76% for strain-accuracy for 25bp fragments.

Wang *et al*. [46] shows reasonable classification of 16S rRNA sequences while Rosen *et al*.'s [64] technique can use any fragment including reasonable performance on short-sequence reads. Because Manichanh *et al*. [74] shows RSR-based classification is advantageous to 16S, Rosen *et al*.'s approach has its advantages, especially since the approach achieves 76% accuracy for ALL 25bp reads at the strain-level. Wang *et al*. verifies that with 16S rRNA sequences, one can get 83.2% accuracy (200bp fragments) and 51.5% (50bp) on the genus-level *via *a leave-one-out cross-validation(CV) test set. For comparison, Rosen *et al*.'s Naïve Bayes classifier (NBC) achieve 95% accuracy for 100bp and 90% accuracy for 25bp fragments on the species-level.

A direct comparison of NBC with BLAST for 25bp fragments is shown in the table:

The 635 completely sequenced microbial genomes, as of Feb. 2008, are still an incomplete representation of extant

**Table:** 

Taxonomic-level Accuracy	BLAST	NBC
Strain (635 genome training data only)	66%	76%
Species (77 strains, 5-fold CV)	89.2% ± 1.9%	90.2% ± 1.2%
Genera (216 strains, 5-fold CV)	86.0% ± 3.5%	66.3% ± 6.3%

diversity, as the microbial sequencing projects grow exponentially. Metagenomic data will produce a significant set of sequences that cannot be assigned to any known taxon, and the question arises how to estimate the number of unknown species. Huson *et al*. show that anywhere between 10% and 90% of all reads may fail to produce any hits [66].

### 4.2. Unsupervised Taxonomic Classification

Unsupervised techniques are usually based on a clustering method, although information-theoretic and text-mining measures have been used [86, 87]. Recognizing that BLAST can only identify a fraction of reads in metagenomics data, clustering has been a natural step [88]. It has been recognized that supervised methods may be insufficient to represent all the extremely diverse microbial genomes. Recently, new methods have emerged to expand the power of unsupervised clustering [89-92]. Chan *et al*. [89] uses Self-organizing maps (SOM) and Growing-SOM (GSOM), which group items based on an adaptive filter learning model, to cluster 1kb to 10kb sequences. Another promising technique is Compostbin, which clusters 6 mer feature vectors (4096 features) of reads based on principal component analysis, and then iteratively segments the data based on a semi-supervised algorithm. On low-complexity datasets, 2-6 genomes per metagenomic sample, the highest error rate was 10%. This approach must now be validated on complex mixtures. In Nasser *et al*. [91], a fuzzy k-means clustering method uses GC-content and different order Markov chains features of two different organisms and genera, which obtains 99% accuracy but still needs to be tested on a more complex mixture. Another promising technique by Li *et al*. uses a similarity-based clustering to form groups that then are matched to known ORFs. Then, a consensus sequence is chosen to represent each family to filter out non-protein-coding ORFs [92]. From this study, 33,000 protein clusters were predicted from the 17.4 million ORFs, and 20% of the predicted ORFs were previously unknown, which might represent novel protein families. While unsupervised clustering techniques remain relatively uncharted territory, these methods hold promise for discovering new organisms and genes in metagenomics datasets.

### 4.3. Methods for Constructing Environmental Community Trees

Each environmental community is composed of a different phylogenetic composition, and there are many different methods for constructing its phylogenetic tree [93]. Generally, each method used for tree construction will lead to a different conclusion of the taxonomy of the organisms under study. However, there is nature's ground truth for the taxonomy of the organisms. Therefore, researchers may employ several models for tree construction for a given set of data. From these multiple phylogenetic trees they attempt to arrive at a consensus of the environment under study [94]. Therefore when performing a comparative metagenomic analysis we are motivated to construct a phylogenetic tree for each environment.

Most phylogenetic reconstruction is based on short subunit 16S rRNA sequences. Operational taxonomic units (OTUs) at the species level are distinguished when the sequences vary more than 3% [95], whereas a genus-level OTU should not have more than 7% sequence variance [96]. Over 200,000 16S rRNA sequences have been collected over the years, which are being used to construct a universal tree [97]. Although extracting and comparing 16S rRNA sequences is the standard way to classify a sample's contents, it is not without its problems. If PCR (polymerase chain reaction) is used, not all rRNA genes amplify equally well with the same “universal” primers. Also, multiple, nonidentical copies exist in various organisms and may lead to overrepresentation of species.

Accurate taxonomic studies for the family and phylum are now within grasp using next-generation sequencing technology [98]. While this technology is not sufficient to sequence the generally accepted 500 bp 16S rRNA sequence for genus and species studies, there is a 400 bp model on the horizon [47]. Also, devices that are capable of sequencing the entire 16S rRNA gene may be available in the near future [33].

Regardless of the sequencing technology used, taxonomists can begin classifying an organism using various analytical statistical tools. Numerous researchers have developed software tools both to aid in the alignment of sequences and tools for developing phylogenetic (evolutionary) trees, all of which can be utilized for taxonomic purposes. Many of these have been incorporated into software packages and source code and are offered online. Some are proprietary and are available for purchase; however, the vast majorities are available for free.

Often, a researcher needs to compare two pieces of genetic information between two different organisms. Currently, a common technique is to align two sequences before any phylogeny can be inferred. The function of sequence alignment between two primary sequences of DNA, RNA or proteins is to determine regions of similarity between the two samples that may identify a structural or evolutionary relationship [99]. Once a relationship has been determined, an evolutionary tree may be constructed.

The software packages highlighted in this section are:

**Table:** 

Purpose	Tool	Algorithm	Access	Cost	Website
Sequence Alignment	BLAST [41]	Local alignment; similar to Smith-Waterman	Server; Executable	Free	*http://blast.ncbi.nlm.nih.gov/Blast.cgi *http://www.ncbi.nlm.nih.gov/blast/download.shtml
	Clustal [100]	Global alignment; distance matrix, neighbor-joining	Server; Executable	Free	*http://www.ebi.ac.uk/clustalw/ *ftp://ftp.ebi.ac.uk/pub/software/clustalw2/
Phylogeny Inference	MEGA [101]	Graphical Clustal ; Parsimony, neighbor-joining, UPGMA	Executable	Free	http://www.megasoftware.net
	PAUP* [102]	Maximum Parsimony	Executable	$100	http://paup.csit.fsu.edu/downl.html
	MrBayes [103]	Bayesian inference	Executable	Free	http://mrbayes.csit.fsu.edu
	Phylip [104]	Parsimony, distance matrix, bootstrapping, maximum likelihood	Executable	Free	http://evolution.genetics.washington.edu/phylip.html
	UniFrac [105]	UniFrac distance metric; P-test	Server	Free	http://bmf.colorado.edu/unifrac

#### 4.3.1. Sequence Alignment

In addition to pairwise alignment methods, Smith-Waterman and BLAST [41], multiple alignment methods can be used to compare multiple sequences at a time and be used for phylogenetic tree construction. The tradeoff is speed and accuracy where global alignment generally takes longer to compare than local, but has great accuracy. Unlike BLAST which uses local alignment, Clustal [100] performs sequence alignment globally, which may be more accurate. However, Clustal should not be used when multiple sequences are entered that do not share common ancestry. This type of alignment is better suited for BLAST, since BLAST compares the sequences against known databases. The Clustal algorithm attempts to align the sequences in query that are most-closely related to one-another to build a representative profile of the family of sequences [106]. Using dynamic programming the basic alignment algorithm consists of three main stages: a) all pairs of sequences are aligned separately in order to calculate a distance matrix giving the divergence of each pair of sequences, b) a guide tree is calculated typically using the Neighbor-Joining method from the distance matrix and c) finally, sequences are progressively aligned according to the branching order in the guide tree.

#### 4.3.2. Inferring Phylogenies

Generally, a phylogenetic tree is created for taxonomic purposes. Each organism on this evolutionary tree represents a node in which these descendants can be traced back to a common ancestor. To build a tree, a researcher first needs to have a file of aligned sequences such as the output files from an alignment method. These files would then be input to various software packages that have been developed for inferring phylogenies to generate the evolutionary tree. The most frequently cited phylogeny packages include PAUP* [102], MrBayes [103], Phylip [104], annd MEGA [101]. A new tool that builds and compares trees from metagenomics datasets is UniFrac [105].

Parsimony is the classical method for building trees using a non-parametric statistical method. Both PAUP* and Phylip utilize this algorithm. Parsimony searches for minimum length trees, i.e. trees that require the least evolutionary change to explain the set of aligned sequences describing them. Additionally, many clustering methods are used as an alternative to parsimony, such as neighbor-joining, Bayesian inference, and UPGMA [107]. MrBayes's use of this approach allows the user to compare heterogeneous data sets consisting of morphological data, nucleotides and proteins in a single analysis. Phylip also invokes maximum likelihood methods and bootstrapping to assign confidence levels to the tree. It is difficult to compare algorithms because taxonomy is constantly changing, and each is used on a different dataset. In addition to parsimony, neighbor-joining, UPGMA and Bayesian inference also have widespread use.

Other methods that use maximum likelihood (ML) method have been well established for phylogenetic tree reconstruction [108-110]. The objective is to maximize the likelihood of the mutation rates between different sequences while simultaneously estimating the tree topology [111]. The evolution between the sequences may be modeled by a discrete-state continuous-time Markov process on a phylogenetic tree. The substitution matrix determines the Markov process. This matrix may be estimated using the expectation maximization algorithm described in [110]. Another substitution model such as Jukes-Cantor may be chosen [112]. The ML method is advantageous in that it provides robustness against incorrect parameter selection in the underlying substitution model [111]. However, model selection is a critical component in a ML phylogenetic analysis and should be carefully considered as the resulting phylogenetic tree could change depending on the model [111, 113]. For large data sets it is computationally expensive to search for the ML phylogenetic tree. Therefore, additional methods such as neighbor-joining are employed to expedite the analysis [110, 114].

There are tools available that enable researchers to compare multiple environmental community trees in a phylogenetic context. UniFrac was developed to analyze significant differences between these multiple environments [105]. To accomplish this it implements the UniFrac significance test and the ubiquitous statistical P-test [115]. Once a researcher has found that there may be a significant difference between two or more environments they can perform a lineage-specific analysis which is also integrated in UniFrac. Using the G-test, a method similar to the chi-squared test for goodness of fit, the tool determines whether particular lineages within a global phylogenetic tree (consisting of all the environments in the comparative analysis) are abundant with sequences from a particular environment [116]. Thus environments may be clustered with respect to consisting of a particular lineage. With Unifrac, it has been shown that humans living in different geographic locations have distinct gut microbiomes.

### 4.4. Microarrays for Organism Detection

Microarrays, DNA chips composed of spots (wells that contain probes), are printed with DNA probes that hybridize with complementary DNA sequences [117]. The probes are short and are designed to unique identify target DNA/RNA sequences. A common use is for the detection of mRNA and gene expression. However, recently, this technology has been extended for organism detection in a given environment, e.g. air, soil or water [118-121]. The traditional caveat of microarrays is cross-hybridization, but it is hypothesized that grouping and compressed sensing methods can minimize and actually leverage information from this biochemical phenomenon [118]. Currently, a large number of probes (and therefore spots) are needed to detect a vast amount of organisms. Therefore, the goal of group-testing and compressed sensing microarrays (CSM) is to reduce the number of spots needed and cost of these devices.

Group testing design was extended by Schliep *et al*. [122] and applied to cover each target with a certain number of probes to allow identification of several targets simultaneously, while using a reasonably small total number of probes. In group testing, a potential group is specified by a probe which hybridizes to a set of target sequences. For instance, a potential target group only exists if there is a probe that binds to all - and exclusively those - sequences in the target. Probe selection for group testing is achieved by an algorithm known as SEPARATE, developed by Schliep *et al*., which avoids cross-hybridization between targets. This method has its disadvantages. For instance, Schliep *et al*. mentioned that out of 19 of the 679 sequences chosen, they were unable to find any suitable oligos demonstrating that the algorithm may fail to find suitable probes. Therefore, microarray target detection can be improved.

In recent years, compressed sensing in signal processing has promised to overcome the lack-of-satisfactory probes from group testing by using fewer probes for organism identification. The essential idea of compressive sensing (or sampling) is that an inherently sparse signal can be recovered by using far fewer measurements than what is typically needed by Shannon's law. Current CSM (compressed sensing microrray) designs focus on: 1) sensing organisms through unique DNA pattern identifiers, rather than single DNA sequences per organism [118], and 2) leveraging cross-hybridization properties of DNA sequences as useful side information for genetic identification [118, 120], and 3) using multiple probes per spot so that the number of spots is significantly fewer than the number of organisms [121].

The compressive sensing DNA microarray is a type of group testing. In CSMs, however, organisms are being grouped according to their DNA sequence similarity. Such groupings are obtained by using the Cluster of Orthologous Genes website (COGs), which organizes prokaryote and unicellular eukaryotes into groups according to the similarity of their protein sequences [118]. Sheikh *et al*. [118] extracted probe candidates from the shortest genes in a group of organisms, thus restricting the full search space and not yielding the optimal probe candidates. Yok *et al*. [120] have introduced an alternative compressive sensing probe picking algorithm, which consider all possible hybridization affinities and chooses the best group identifier probe among all possible probe candidates from all the members of a group [120].

## 5. GENE-CENTRIC METAGENOMICS: FUNCTIONAL CLASSIFICATION OF SAMPLES

Beyond asking “who” and “how many,” the next question is “What are they (the microbial communities) doing?” By using high-resolution community-wide genomic information, we can describe the composition, function, and emergent properties of integrated microbial communities more accurately. Such analyses might distinguish the characteristics associated with environmentally-robust bacterial communities from those that allow pathogens in certain habitats.

In fact, several recent gene-centric studies have focused on comparative metagenomics to investigate whether distinct commonalities and/or differences can be observed in microbial communities that can be attributed to their habitat or physical environment. The consensus opinion of these studies indicate that there is a strong correlation between the communities and the habitat in which they live, whether the environment is soil, marine or the human gut. Tringe *et al*. (2005)'s seminal work [23], for example, compared samples from agricultural soil, deep-sea whale-fall carcasess, the Sargasso Sea and the acid mine drainage environments. Using a clustering based approach, they showed that profiles of the microbial communities from each environment clustered with those of others in the same community, and concluded that “functional profile of a community is influenced by its environment.” Similar comparative analyses have also shown the existence of “functional anchors in complex microbial communities” of the human gut [123], or that while some rare members of the soil bacterial community were closely related to abundant taxonomic groups, a significant portion of the “rare biosphere showed evolutionarily distinct lineages at various taxonomic cutoffs” [124]. Fierer *et al*. [22, 125] compared the diversities, richness and evenness of four major microbial taxa, (bacteria, archaea, fungi, and viruses), in prairie, desert, and rainforest soils, concluding that all communities display local as well as global diversity. The same group also showed that bacterial diversity was unrelated to physical features (such as temperature) that typically predict plant and animal diversity, however, the diversity and richness of soil bacterial communities does differ by ecosystem type. Allison *et al*. investigated whether microbial community composition is resistant, resilient, or functionally redundant in response to different environmental disturbances (and concluded that they are not) [126]. On the other hand, Kurokawa *et al. *showed that gut microbiota from unweaned infants were simple with a higher variation in taxonomic and gene composition, while those from adults and weaned children were more complex with a higher functional uniformity regardless of age or sex [14]. De Long *et al*. compared microbial communities from the ocean's surface to near-sea floor depths, which showed “vertical zonation of taxonomic groups,” suggesting “depth-variable community trends in carbon and energy metabolism,” among other interactions [127].

While the aforementioned studies established that there is a relationship between functions of communities and their habitats, a separate line of work tried to determine exactly what those functions are. An important first step to discern function is to find the regions of DNA which encode for proteins. Early gene finding methods focused on finding Open Reading Frames in DNA sequence. An Open Reading Frame is generally defined as a sequence of DNA that begins with a start codon and ends with one of the stop codons. Many methods have been developed for locating ORFs within a DNA sequence, including simply locating start and stop codons, as in the NCBI ORF finder tool [128]. This simple method, however, only gives us ORFs but does not indicate which regions actually encode proteins. Methods such as GENIE [129], GENSCAN [130], GENEMARK [10], GLIMMER [131], not only look for regions with start and stop codons but also predict whether the region in question has a chance of actually encoding for a protein. GENIE uses a generalized HMM to give a gene model of a DNA sequence [129].

GeneMark [10] or GLIMMER [131] can be used to predict protein coding regions in prokaryotic organisms. It scores coding regions by creating an HMM with 9 hidden states. GLIMMER, on the other hand, improves on GeneMark by using interpolated Markov models (IMMs) with varying orders (instead of the fixed 5th order HMM used by GeneMark) [131]. Specifically, Glimmer uses models ranging from 1st through 8th order and combines three periodic-nonhomogeneous Markov models in the IMM to predict protein coding regions. In metagenomic samples however, most bacteria and their genes have not been previously sequenced, resulting in little training data being available for these training-reliant methods. Thus a set of new methods must be developed in order to perform gene finding on previously uncultured environmental samples.

### 5.1. Towards Functional Metagenomics

#### 5.1.1. Metagene [132] 

MetaGene is a utility that seeks to make use of existing packages on the web to analyze predicted gene features. MetaGene uses a large set of prokaryotic genes in Genbank [133] to create a training set, and runs in two stages. First, all ORFs are extracted from the data and are scored according to their base compositions and lengths. Partial ORFs are only extracted if they encompass the entire sequence being analyzed, or if they appear at the very end of a sequence. The second stage uses these scores, as well as the distances of neighboring ORFs, to find an optimal combination of ORFs. Metagene's computes its scores using log-odds ratios on such features as di-codon frequency, ORF length distributions, distance distributions from an annotated start codon to the nearest start codon and frequencies of orientations and orientation dependent distances of neighboring ORFs [132]. MetaGene was first tested on whole bacterial genomes and compared to GeneMark, which unlike MetaGene, uses CG% to estimate codon frequencies and distance distributions and performed comparably for the bacterial and archaeal genomes analyzed in the test. On the other hand, while performing well on long shotgun sequences, no performance analysis is shown for shorter reads, and there has been no significant investigation for hypothetical gene regions identified by GeneMark. Therefore, the feasibility of this approach for finding novel genes is currently unknown.

#### 5.1.2. Harrington et al. [134]

While MetaGene shows promising results when known genes are used as a training set, it only evaluates regions based on simple criteria and it has no ability to predict function. Harrington *et al*. propose an approach that analyzes ORFs to infer function from the proteins these regions coded for [134]. Harrington *et al*.'s method was evaluated on Genbank as well as other functional databases such as KEGG [135], COG [136], UniRef [137], SMART [138], and Pfam [139]. Specifically, Harrington *et al*. use these databases to find gene regions inside environmental samples with high similarity, or in the domain or gene neighborhood as existing protein sequences. The approach allows categorizing the ORFs as being in the domain of known proteins even though many of the bacteria in these environmental samples have never been cultured. This means that the ORF regions with little or no similarity to known sequences may be inferred as being in the same family or domain as a group of known proteins. By using a combination of functional and sequence similarity along with genomic neighborhood, Harrington *et al*. were able to infer function for 76% of the ORFs found in four different environmental samples. Previous to this study, function was only predicted for 27%-48% of the ORFs in three different wale fall carcasses [134]. It should be noted, however, this method has only been demonstrated to work on longer sequence reads.

#### 5.1.3. Yooseph's Incremental Clustering [140]

Clustering approaches can also find gene regions and identify their functions. One such method uses known protein families and sequences as inputs to identify protein coding regions, and cluster the data based on their function [140]. This method was compared to MetaGene and was found that a large portion of the identified regions overlapped. Of those regions that did not overlap, only 4% of the MetaGene predictions had matches to Pfam models, as opposed to 21% with the clustering method. Yooseph's method was also shown to have high specificity, though its sensitivity in detecting a gene is dependent on the representation of existing protein clusters in the organisms' neighbors (taxonomic).

#### 5.1.4. Hoff et al. [141]

Many of the aforementiond methods have difficulties dealing with shorter fragment lengths produced by pyrosequencing. To address this issue, Hoff *et al*. developed a two-stage machine learning approach to gene prediction that analyzed performance for fragments ranging in size from 100bp to 2000bp. First, linear discriminants are used to extract features from identified ORFs. Incomplete ORFs are permitted as many ORFs could be fragmented due to pyrosequencing. The features extracted are monocodon and dicodon usage, translation initiation sites, ORF sequence length, and CG content. In stage 2, these features are used to build a multilayer perceptron (MLP) neural network for binary ORF classification (coding or non-coding). The trained MLP then determines the final coding candidates. The authors note their results to be similar to MetaGene, and conclude that their method's ability to have high prediction specificity complements MetaGene's high sensitivity. Therefore, they recommend a combination of the two methods for gene finding in metagenomic samples [141].

The method's benefit is that it directly addresses relatively short fragments. It does not however attempt to infer the function of any of the predicted genes or to group those genes based on their potential to have the same function. This could potentially be addressed by combining this approach with that of Harrington's [134].

#### 5.1.5. Dinsdale et al. [142]

Dinsdale *et al. *looked at the possibility that different environments may have different metabolic profiles [142], which was tested using canonical discriminant analysis (CDA). Also known as multiple discriminant analysis or discriminant factor analysis, CDA seeks to classify cases into three or more categories using dummy categorical variables as predictors. The authors wished to find metabolic functions (the variables in CDA) that would distinguish different organisms. Samples were sequenced using pyrosequencing and were compared to functional genes in the SEED platform (http://www.theseed.org) using BLASTX with an E-value < 0.0001. In order to perform the CDA the sequences were grouped according to their SEED classification. CDA builds a model for each membership in each group and calculates a discriminant value for each metagenomic fragment (sample). CDA is advantageous because it can identify which variables best separate the groups, analyze those variables only, and discard the rest. The CDA was performed on 15 million sequences from 45 microbiomes and 42 viromes. Most of the variance between the different environments (79.8% of the combined microbiome and 69.9% of the virome) was explained in this analysis, showing that metagenomes are highly predictive of metabolic potential within an ecosystem. In contrast, a recent analysis of 16S rRNA genes from multiple environments only explained about 10% of the variance [143], which suggests that taxa alone is not sufficient, but metabolic function is also needed to distinguish different ecosystems.

#### 5.1.6. Krause et al. [144]

In order to overcome the short-read limitation of next-generation sequencing, Krause *et al*. follow a four-stage approach: First, a BLAST search divides the sequence into six reading frames. BLAST searches are conducted on the amino acid level where each hit is associated with a specific reading frame in the contig. BLAST hits are filtered to retain those indicating the presence of a coding sequence. In stage two, combined scores are calculated which indicate the coding potential of each nucleotide in a contig. The sequence of each reading frame is compared with all the database matches that were generated from the BLAST search prior. The number of synonymous substitutions for each match is used as a positive score with non-synonymous substitutions counting as negative scores. The scores for each position and reading frame are stored in a matrix giving a position specific score that the contig is coding (or non-coding) in one of the six reading frames. In stage three, this matrix is used within a dynamic programming based optimization algorithm to find an optimal path. Finally, in stage four, postprocessing combines predictions from previous steps and identifies frame shifts. This algorithm is computationally expensive due to the dynamic programming, but it achieves good success and is able to quickly process the large number of sequences generated by 454 pyrosequencing.

## 6. BIOMOLECULAR DYNAMICS IN MICROBIAL COMMUNITIES

The main thrust of our review is the analysis of DNA sequence data. However, characterizing the organisms and genes present in a metagenomic sample only tells us the “parts list” of the organisms within the microbial community. Under different environmental conditions and stresses -- such as the presence of toxins or changing nutrient levels -- different parts will be expressed as needed for the organisms within the community to adapt and grow. Furthermore, while sequences that are identified as hypothetical genes based on homology analysis may be found within a metagenome sequence, they may contain mutations or be otherwise non-functional within the microbes that are present in the community. Thus, after sequencing the DNA of a microbial community, we need to understand how the community behaves by identifying what genes are expressed and produce proteins that perform cellular functions. To do so, biological researchers are taking advantage of “post-genome” technologies [117] that were initially developed to analyze the molecular behavior at the level of mRNA molecules transcribed from genes, proteins that are translated from mRNA, and other molecules that are significant for cellular functions. While our review emphasizes signal processing methods applied to metagenome data, we will briefly discuss new applications of technologies to elucidate the dynamics of biomolecular networks that respond to environmental changes: specifically, changing the expression of genes, the level of proteins that are produced, and the levels of metabolites (small molecules) that change with the activity of metabolic pathways within microbial cells.

### 6.1. Metatranscriptomics

Functional genomics is the high-throughput generation of data for the expression of genes in cells. Gene expression is the transcription of DNA to produce mRNA, which goes on to form the template for protein generation. There has been substantial work done on developing platforms to mRNA levels expressed from the whole genome from cells of single organisms. These techniques can be applied to multiple organisms in a community as reviewed in [145], but with an increase in the necessary complexity. One approach is to extend microarrays, which typically have oligonucleotide probes that can identify the presence of mRNA expressed from each gene of a genome. This can be done by developing a microarray that has probes for genes from multiple genomes, such as was done in [146] for the study of 4 microbial species cultured together. However, this strategy requires knowing *a priori* what organisms will be present in a sample or else selecting only a few organisms within a community to study. As an alternative, a microarray can be developed to analyze genes within a set of functional pathways, such as those involved in contaminant degradation [147]. In this strategy, microarrays are designed with probes that recognize regions of these genes that are highly conserved between species [148]. Consequently, the expression of genes with these functions can be detected from many different organisms (including those with unknown organisms). This kind of microarray was recently used to compare gene expression in samples from different ecological niches of Antarctic soil [149].

In general, the microarray platform is limited by the increased cost of adding increased number of probes, as well as the potential for cross-hybridization noise when trying to differentiate between the expression of genes with highly similar sequences. Another strategy that has been employed is high-throughput DNA sequencing technologies employed for metagenomics studies, such as pyrosequencing technology. The mRNA expressed by a microbial community can be isolated and chemically copied to form a complementary DNA strand, which can then be sequenced. This approach has been recently used to analyze gene expression in oceanic samples [150, 151]. Notably, at least 99.9% of the RNA was found to be mRNA expressed from genes, as opposed to ribosomal RNA. Furthermore, in both studies, they found many more genes in the mRNA complement then in a simultaneous sequencing of the DNA isolated from the sample, including approximately 50% of previously unknown genes found by [151].

Like metagenomic DNA sequences, functional metagenomic mRNA data sets represent a large-scale analysis problem. Previous studies have demonstrated the efficacy of signal processing methods for the analysis of gene expression data for single organisms, as reviewed in [152, 153]. These methods include single value decomposition for identifying groups of genes that are expressed under different stimuli [154], unsupervised clustering methods [155], and other pattern recognition methods reviewed in [156]. The analysis and interpretation of gene expression data is still an area of ongoing research. It is reasonable to expect that metagenomic samples will pose new challenges, since many more genes are present in data sets, e.g., 330 million base pairs and potentially 10^5^ genes found by [150].

### 6.2. Metaproteomics

While the mRNA expression of genes drives changes in protein levels under different environmental conditions and stimuli, protein expression dynamics are further regulated by different rates of degradation, post-translational modifications, etc. that cannot be measured with functional metagenomics. The high-throughput measurement of protein expression within a microbial community is called *metaproteomics*, and has been reviewed in [51, 157]. One of the initial studies, which used mass spectrometry (MS)-based proteomics along with metagenomic DNA sequencing, studied a low complexity biofilm from underground mine sites [158]. Further examples of MS-based metaproteomics include the analysis of samples from chlorobenzene-contaminated sites [55], studying uncontaminated soil samples cultured in the presence of cadmium to measure the temporal response of a community to a controlled stimulus [54], and the analysis of a bioreactor used to optimize sludges for phosphorus removal [159]. Besides studying biomolecular dynamics, metaproteomics can also be used to complement the identification of genes and genomes within a community, through directly sequencing peptides (protein fragments) found in samples in an initial MS analysis. This was integrated with DNA sequencing to characterize previously unknown proteins in [55], as well as to distinguish between the expression of proteins from related organisms that differed by as little as a single amino acid in [160] -- a difference so small that sequence analysis would be unable to distinguish the genes that code for them.

As with functional genomics, signal processing methods are critical for the analysis of metaproteomic data. Unlike gene expression data, proteomics data does not cleanly identify the levels of individual proteins. Rather, the mass spectrum of protein fragments is obtained, and peaks are correlated with a database to identify individual proteins. Clustering and other statistical signal processing approaches to this problem are reviewed in [161, 162]. A specific analysis of statistical classification, including various methods based on univariate statistics and principle components analysis, has been reported on representative data sets [163]. Other work has described the use of support vector machines for protein identification and classification [164], as well as the use of FFT for data noise reduction followed by Bayesian clustering on reconstructed data sets to identify proteomic differences between samples [165]. Machine learning methods for proteomics are reviewed in [166], including the application of peak clustering and wavelet-based methods for mass spectrum pre-processing, and the use of classifier methods for identifying proteins that change under different conditions.

### 6.3. Meta-Metabolomics

The principal activity of a microbial cell is to metabolize nutrients and generate energy required to survive and grow. The enzymatic reactions for metabolism are structured in metabolic pathways and networks within a cell. Metabolism in a microbial community is interactive -- the products of metabolism from one species may enhance or inhibit metabolic pathways in other species. And, in a community hosted with a multicellular organism, such as the microbial community in the human gut, metabolic pathways within bacterial cells may interact with pathways within host cells. Changes in the activity of metabolic pathways is reflected by changes in the levels of small molecules that are the substrates and intermediates of enzymatic pathways. The levels of many metabolites can be measured simultaneously through nuclear magnetic resonance (NMR) spectroscopy, reviewed in [167] or by liquid chromatography separation followed by mass spectrometry to identify metabolites by their masses and charge levels, reviewed in [168]. Notably, these *metabolomic* (also known as *metabonomic* in some literature) technologies are inherently “meta-metabolomic” -- measurements of metabolites in a sample from mammalian blood or urine, for example, will reflect the contributions of both the host metabolic pathways as well as those of microbial communities colonizing it.

## 7. METAGENOMICS DATABASES, TOOLS, AND BENCHMARKING

One of the first extensive metagenomics datasets was published in 2004 by the Craig Venter Institute, which composes approximately 2 million reads, averaging 818 bp per read, sampled at 7 different sites in the Sargasso Sea [69, 169]. Sargasso sea analysis countered traditional views that the salty Sargasso Sea is nutrient poor and showed that reads aligned to a diversity of life.

**Fig. (2):**
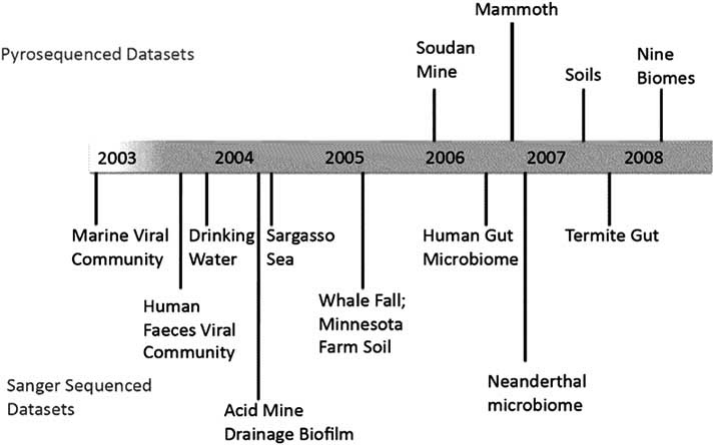
The first metagenomics dataset was shotgun, *via* the Sanger method, sequenced in 2003. Since then, pyrosequencing is now being used to gain cheaper and highly parallel reads. The timeline illustrates some metagenomics datasets that have been sequenced to date and is a subset of all the projects that are completed [40].

Subsequently, many projects have been sequenced and are publicly available (see Fig. **2** for a history). After the Human Gut Microbiome dataset [170] was released in 2006, the NIH (National Institute of Health) made the human microbiome a part of its roadmap initiatives in 2007 [12, 171]. In 2007, the Department of Energy's Joint Genome Intiative (DOE/JGI) had sequenced about 50% of the metagenomics projects including various soil microbiomes, human, mouse, and termite gut samples, and also airborne samples [172, 173]. San Diego State University's SCUMS (SDSU Center for Universal Microbial Sequencing) contains samples from coral reefs, Soudan mine, human lungs, etc. [174]. In 2007, microbes were isolated from the human mouth that come from a previously unknown phylum, TM7 [175]. Because of horizontal gene transfer and possible contamination, some of the genes aligned to the Leptotrichia species. Thus, while it was intended as a single cell genome sequencing project, the result is considered a metagenomic dataset [176].

Some of the databases online provide their own tools for analysis. Two of such online services are CAMERA (Community Cyberinfrastructure for Advanced Marine Microbial Ecology Research and Analysis) [177, 178] and the MG-RAST (Meta Genome Rapid Annotation using Subsystem Technology) [179] server. Much of CAMERA's tools are visualizations of the BLAST hits of the reads. The tools included in RAST are annotation, phylogeny, metabolic reconstruction and visual comparison tools.

With the vast amount of data becoming available and published, researchers are calling for a standardization process to register new projects, tools, and other publications [180]. There is also contamination present in some of the metagenomics datasets such as in the Sargasso Sea dataset [181]. Also, metagenomic datasets contain many unknown phyla, genera, and species. If a standardized metagenomics dataset is designed to simulate training and test data, computational tools can use such a dataset to benchmark and compare their performance for known and unknown organisms. The first such attempt at simulating metagenomic data has been released and is called MetaSim [182].

## 8. FUTURE APPLICATIONS

As metagenomic approaches become more feasible and cost-effective, we stand to gain a large amount of sequence data from previously uncultured and uncharacterized microbes. The expected influx of these data will undoubtedly shed a great deal of insight into the bacterial phylogeny, enabling us to study the evolution of many novel lineages that live in complex communities within previously understudied environments. Two applications that are of interest are health diagnosis and food security that we present in this section.

### 8.1. Correlation of Metagenome to Function for Obesity

As metagenomics and metaproteomics advance, the pivotal process in the field will be to merge the two and infer collective function from the interactions of multitudes of microbial species. One important example applies to human health in a recent study by Turnbaugh and colleagues [183]. Using a combination of 454 and Sanger sequencing, the authors sequenced the metagenome of lean and obese mouse littermates. After performing a functional annotation of the sequenced fragments, genes were classified into distinct functional categories. The relative abundances of sequences from these categories were then compared between lean and obese siblings to identify differences in the genomic signatures of their distal gut communities. Strikingly, their analyses illustrated that gut microbes from obese mice were enriched for genes encoding enzymes that metabolize “indigestible” polysaccharides. Combined with experimental evidence from caloric measurements of mouse feces, this indicated that the gut bacteria of obese mice are better able to extract energy from their hosts’ diets, providing a plausible means by which bacteria could promote obesity. Accordingly, Turnbaugh and colleagues demonstrated that the addition of “obese” microbial communities to germ free mice did indeed lead to an increase in body fat.

Several observations reveal that these findings have direct implications for obesity in human populations. First, analyses of 16S rRNA sequences reveal that bacteria from the phylum Firmicutes are more abundant in the guts of both obese mice and humans compared to the guts of their lean conspecific counterparts [11, 184]. Second, and conversely, bacteria from the phylum Bacteroidetes were less abundant in the guts of obese mice and humans compared to the guts of lean individuals [11, 184]. Third, and most importantly, human weight loss was correlated with a concomitant decrease in Firmicute bacteria and a corresponding increase in the proportion of “healthy” Bacteroidetes [11]. So combined, these findings implicate bacteria as playing a direct role in human obesity, identifying novel targets in the fight against this growing epidemic.

### 8.2. Food Security

An example of a future linkage between metagenomics and function is soil microbial community assessment for agricultural decision making and food security. The presence in soils of specific plant pathogens, pests, growth inhibitors, and nutrient imbalances can interfere to unknown degrees with the production of desired crops. The absence in soils of specific plant symbionts or root associates, on the other hand, can also limit crop productivity. Soil metagenomics offers the means to diagnose functional capabilities of microbial communities for optimizing agricultural production on arable lands, the supply of which is becoming more limited in the face of a rapidly growing global population. Unbeknownst to us today, soils may not be providing optimal yields due to the lack of microbial assemblages needed for improved plant growth or disease resistance, despite provision of adequate fertilizers and appropriate cultivation practices. Moreover, current agricultural practices, such as fertilization with animal manures or municipal biosolids, may foster the establishment of soil microbial communities that pose food safety threats by serving as reservoirs for emerging pathogens or by facilitating exchange of antibiotic resistance genes among microorganisms [27]. Thus insights from linking metagenomics and function can help improve the safety and sustainability of our food supply.

Greater understanding of microbial communities and the factors that drive their compositions will be key in engineering better human health, food security, and environmental quality. While still at an early stage, these findings highlight the utility of metagenomics in studies of human disease, soil productivity, and ecosystem services, while also revealing a new-found ability to elucidate and compare genomic signatures of natural bacterial communities.

## Figures and Tables

**Fig. (1):**
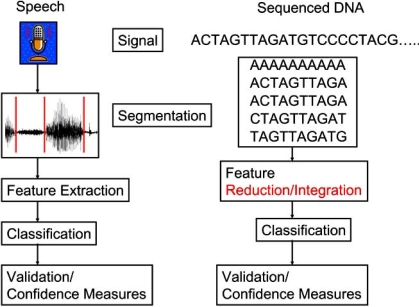
Comparison of Speech Classification to the DNA Classification problem.
